# Modulation and control of transformerless boosting inverters for three-phase photovoltaic systems: comprehensive analysis

**DOI:** 10.1038/s41598-025-93902-9

**Published:** 2025-04-23

**Authors:** Mostafa Wageh Lotfy, Ramadan Mahmoud Mostafa, Haitham S. Ramadan, Mahmoud F. Elmorshedy, Sherif M. Dabour

**Affiliations:** 1 Department of Process Control Technology, Faculty of Technology and Education, Beni-Suef University, Beni Suef, Egypt; 2 Electrical Power and Machines Department, Faculty of Engineering, Zagazig University, Zagazig, 44519 Egypt; 3 ISTHY, Institut National du Stockage Hydrogène, 90120 Morvillars, Belfort Territory France; 4 Renewable Energy Lab, College of Engineering, Prince Sultan University, Riyadh,11586, Saudi Arabia; 5 Electrical Power and Machines Engineering Department, Faculty of Engineering, Tanta University, 31733 Tanta, Egypt

**Keywords:** Split source inverter, Voltage source inverter, Photovoltaic (PV) system, quasi-Z-source inverter (q-ZSI), Electrical and electronic engineering, Energy infrastructure

## Abstract

This paper examines the performance of three power converter configurations for three-phase transformerless photovoltaic systems. This first configuration consists of a two-stage DC–DC–AC converter comprised of a DC–DC boost chopper and a three-phase voltage source inverter. The second and third configurations are the single-stage quasi-Z-source inverter (qZSI) and the split-source inverter (SSI). The performance of the presented topologies has been analyzed and compared in terms of topological requirements, modulation techniques, and control of output voltage, considering both ideal and parasitic cases. Moreover, the voltage and current stresses on the devices, passive elements, and efficiency are also addressed. Simulation and experimental testing were subsequently carried out to validate the analysis and evaluate the performance of the proposed topologies.

## Introduction

**Fig. 1:**
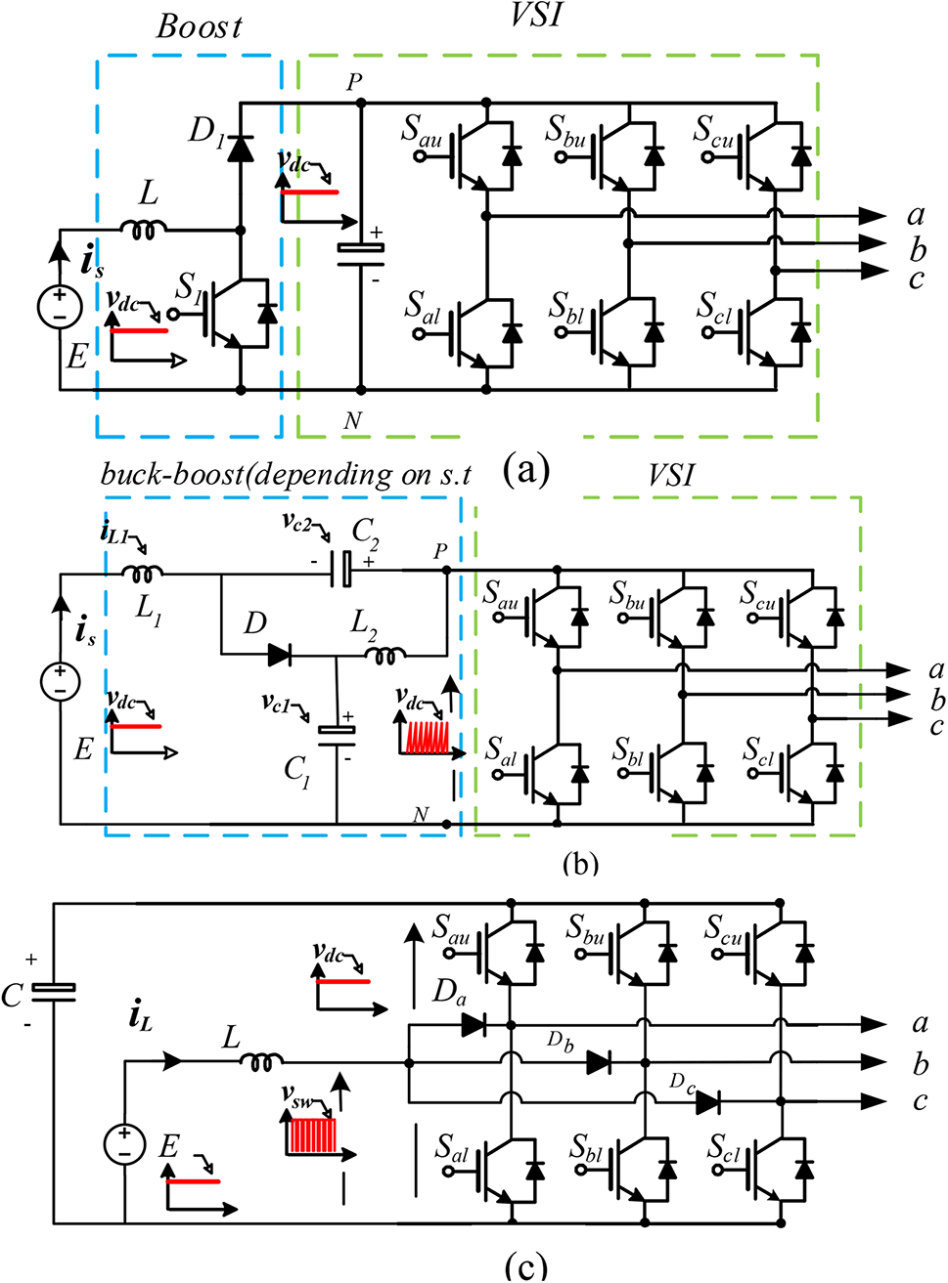
Analyzed topologies—(**a**) two-stage boost inverter, (**b**) quasi-Z-source inverter (q-ZSI), and (**c**) split-source inverter (SSI).

VOLTAGE-SOURCE INVERTERS (VSIs) are the most widely spread dc–ac power converters. However, VSIs only allow for dc–ac inversion with buck capabilities, i.e., the output AC line voltage of conventional inverters is inherently constrained by the magnitude of the available DC input voltage, which poses a significant limitation for their application in renewable energy systems. This constraint becomes particularly problematic in photovoltaic (PV) and fuel cell-based systems, where the input voltage is often low, variable, and subject to fluctuations due to changes in environmental conditions such as solar irradiance or fuel cell dynamics. As a result, these conventional inverter topologies are generally considered unsuitable for direct integration with such energy sources, as they fail to provide the necessary voltage boosting capability and stability required for efficient and reliable power conversion^1,2^. This challenge underscores the need for advanced inverter topologies, such as Z-source or quasi-Z-source inverters, that can simultaneously perform voltage boosting and inversion in a single stage, thereby enhancing the efficiency and adaptability of renewable energy conversion systems. The addition of a boosting stage has thus commonly been used to increase the dc-voltage level, as clarified in Fig. 1a^3^.

Z-source inverters (ZSIs) that provide boosting and inversion in a single stage have recently gained attention owing to their reduced size, cost, weight, and system complexity compared with their two-stage equivalent^4,5^. The ZSI topology serves as the foundational circuit for many recently developed single-stage topologies. Over the past two decades, more than 2000 publications on this topic have appeared in the IET and IEEE databases. Key milestone concepts in this field are comprehensively detailed in references^6–10^.While the basic ZSI’s core purpose remains the same, its topology, modulation, boosting capability, reliability, and efficiency have been improved^7^. The first improved topology is the quasi-Z-source inverter (q-ZSI), shown in Figure 1b^11^. The structure of the q-ZSI is very similar to that of the basic ZSI, except that the input current is continuous, and the voltage stresses are lower^12^. It is commonly used for single-stage PV systems owing to its continuous input current and simple structure^13,14^. Many applications, including automotive and renewable energy systems, have also used qZSI^6,15^. In the three-phase quasi-Z-source inverter (q-ZSI), an impedance network consisting of two inductors, two capacitors, and a fast-recovery diode is strategically placed between the input DC source and the conventional B6 bridge. This network plays a critical role in enabling the inverter’s voltage-boosting capability. Unlike traditional inverters, the q-ZSI introduces shoot-through (ST) states alongside the standard eight switching states to achieve this boost in voltage. The ST states are generated by employing seven unique combinations of the B6 bridge, which allow the inverter to momentarily short-circuit the bridge, thus storing and releasing energy from the impedance network to elevate the output voltage. This innovative approach not only enhances the inverter’s voltage range but also contributes to its resilience against input voltage fluctuations^7^. Despite this, the qZSI has been laboratory-confirmed to perform well in PV and FC systems. Nevertheless, it appears that it cannot be substituted for the two-stage architecture due to the need for a longer shoot-through time to obtain a high voltage gain, affecting the system’s efficiency and output voltage quality^16^.

There have been several modulation schemes proposed to enhance the boosting capability. These include maximum boost control, constant boost control, maximum constant boost control, and modified space vector modulation. These schemes are compared and evaluated in^17^.

It should be noted that ZSI/qZSI work within a limited modulation index range as (0.5 < M < 1), The modulation index and output voltage gain have an inverse relationship, meaning that as the modulation index decreases, the output voltage gain increases. However, this inverse relationship leads to higher Total Harmonic Distortion (THD) in the output voltage and current waveforms when a high gain is required^18^. Among the shortcomings reported in^19^ that limit their application in the industry are:

- The sum of the M and ST ratios is constant and limited by 0.5. Consequently, a low value of M is chosen for a high boosting ratio. This reduces the ac voltage quality.
- The DC link has a pulsating voltage.

The split-source inverter (SSI), illustrated in Fig. 1c, is a relatively new topology that has emerged by integrating a DC-boost converter directly into the traditional three-phase voltage source inverter (VSI)^19–25^. This innovative design connects the boost inductor to the AC output terminals of the inverter legs through three diodes, enabling the inverter to achieve both voltage boosting and inversion within a single stage. This integration simplifies the overall circuit design by reducing the need for separate boosting stages, enhancing efficiency and compactness. The SSI topology is particularly advantageous in applications where space and efficiency are critical, as it consolidates the functions of voltage boosting and AC power generation into a streamlined configuration. The boost converter was first applied to dc–ac power conversion by Caceres and Barbi^8^ and Abdelhakim et al.^9,10,13,26–34^, The earlier approach focused on integrating two boost converters to achieve a sinusoidal output voltage. In contrast, Split-Source Inverters (SSIs) offer a more efficient design by utilizing fewer passive components compared to quasi-Z-source inverters (q-ZSIs). SSIs maintain a continuous input current, which is beneficial for reducing input ripple and improving overall stability. Additionally, SSIs can employ the standard modulation strategy that utilizes the eight conventional states of a Voltage Source Inverter (VSI), simplifying the control process. One of the key advantages of SSIs is their ability to maintain a constant DC-link voltage with minimal low-frequency components, which enhances the quality of the output voltage and reduces the stress on the power switches, thereby improving the overall efficiency and reliability of the system. This makes SSIs an attractive option for applications requiring a compact, efficient, and robust power conversion solution.

Despite significant efforts by researchers in this field, comprehensive analyses focused on switching losses have not garnered the same level of interest^10^. This work, therefore, aims to review the three transformerless topologies, including the two-stage boost inverters, q-ZSIs, and SSIs, compare their topologies, and evaluate their performance and suitability for PV applications. Moreover, a comprehensive analysis of topological requirements, modulation techniques, output voltage control, and current stresses is performed. This work is organized as follows.

In “Analyzed topologies” section, the three topologies are described. A review of the available topologies and their modulation techniques, voltage gain, and boosting action control methods are introduced in “Comparison items and equations” section.

Finally, the simulation results and experimental validations are discussed and followed by conclusions.

## Analyzed topologies

The three analyzed topologies, shown in Fig. 1, can be classified as two-stages topology and one-stage topology.

The two-stage topology depicted in Fig. 1a consists of a standard DC-DC boost chopper followed by a three-phase Voltage Source Inverter (VSI). In contrast, the quasi-Z-source inverter (q-ZSI), shown in Fig. 1b, functions as a DC/DC buck-boost converter, depending on the shoot-through (ST) ratio, with key components facilitating DC/AC conversion. The q-ZSI operates in two distinct modes: non-ST and ST^10,26^. In non-ST mode, it functions similarly to a traditional VSI. However, in ST mode, at least one leg of the inverter is intentionally short-circuited to enable the voltage boosting action.

The q-ZSI achieves this boost without the need for additional active switches, leveraging the B6 bridge to generate the ST states. During ST mode, the zero state vectors of the inverter are partially or entirely utilized to amplify the input DC voltage ${E}_{s}$. This voltage amplification is primarily achieved through three specific ST control techniques: simple-boost (SB), maximum-boost, and maximum-constant-boost (MCB). These techniques are crucial for optimizing the q-ZSI’s performance, allowing it to efficiently convert and boost voltage within a single integrated stage^27^.

The recently proposed SSI topology, given in Fig. 1c, can satisfy the boosting action with the minimum number of reactive elements, in which only one inductor and one capacitor, as in the two-stages case, are used. However, it requires additional three power diodes. SSIs have two modes of operation, inductive charging and inductive discharging^35–40^.

## Comparison items and equations

**Fig. 2:**
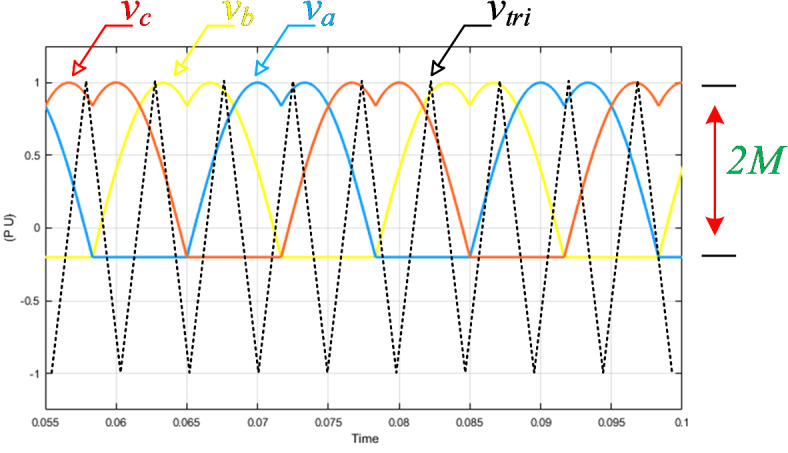
Three-phase modulating and carrier signals of the MSVM scheme.

To obtain a fair comparison, the inverter stage (B6 switches) of the traditional two-stage dc–dc–ac converter, the qZSI, and the SSI are modulated via the modified space vector modulation (MSVM) schemes, in which the modulating signals are given in Fig. 2^28,29^.

Parameters vital for an inverter analysis are listed below.

$${V}_{dc}$$
The input voltage to the B6 bridge.
$$\cos (\Phi )$$
Power factor.
$$K$$
Boosting factor.
$$M$$
The modulation index of inverters.
$$\widehat{V}$$
Ac peak phase voltage or output peak voltage of the inverter.
$$\delta$$
The average duty ratio of the ST-state, equal to ${T}_{sh}/{T}_{s}=\left(2\pi -3\sqrt{3}M\right)/2\pi$

### Topology

This section provides a comparative analysis of the different topologies based on the number of active (semiconductor) and passive components involved. In the two-stage topology, an extra boosting stage is implemented to elevate the input DC voltage before it is converted to AC using the traditional B6 bridge inverter. This configuration necessitates the inclusion of an additional active switch and a forward diode, along with a single inductor (L) and a capacitor, to facilitate the necessary voltage boosting^41^. As a result, this architecture comprises a greater number of components, which can influence both its complexity and overall efficiency. By examining the component count, this comparison highlights the trade-offs between the various topologies in terms of design simplicity and performance capabilities.

The q-ZSI performs the boosting action using the B6 switches without any additional active elements to perform the ST operation. However, a q-ZSI employs two inductors (L_1_ and L_2_) and two capacitors (C_1_ and C_2_) in its impedance network.

To perform the boosting action alongside dc–ac inversion stage, additional switching states outside the conventional eight states of the standard VSI are used to obtain the ST operation.

The Split-Source Inverter (SSI) employs the same B6 bridge configuration as the traditional three-phase Voltage Source Inverter (VSI), utilizing the standard eight switching-state combinations. In this setup, at least one of the lower switches (${S}_{al},{S}_{bl},{S}_{cl}$) is activated to facilitate the charging of the inductor L. This approach not only simplifies the design by maintaining compatibility with established VSI techniques but also enhances the inverter’s efficiency by effectively managing energy storage within the inductor. By leveraging these standard switching states, This operation can be performed via seven switching-state combinations (${V}_{0}$–${V}_{6}$). However, the remaining switching state ${V}_{7}$ is used to discharge the inductance $L$ through the dc-link capacitor $C$, The q-ZSI has several significant drawbacks. Firstly, the shoot-through duty ratio must always remain below 0.5, which limits its effectiveness in applications with very low input DC voltage. Additionally, the use of high-voltage capacitors is necessary, which contributes to increased costs and larger system size.

From the discussion mentioned above, the following conclusions can therefore be made:

- The SSI and q-ZSI configurations allow for boosting the input dc voltage as in the two-stage configuration.
- Both the SSI and q-ZSI topologies demand a greater number of active switches and diodes for the charging and discharging of the inductor compared to the conventional two-stage solution. This increased component requirement results in higher current stresses on the switching devices, leading to greater conduction losses.

### Modulation

To get a fair comparison between the modulation schemes of the presented topologies, a high-frequency triangular carrier, ${v}_{tri}$ is compared with the modulating signals of each to generate the switching pulses. As shown in Fig. 2, the modulating signals are given by injecting a third-harmonic signal to the sinusoidal references to achieve the maximum possible modulation index.

**Fig. 3:**
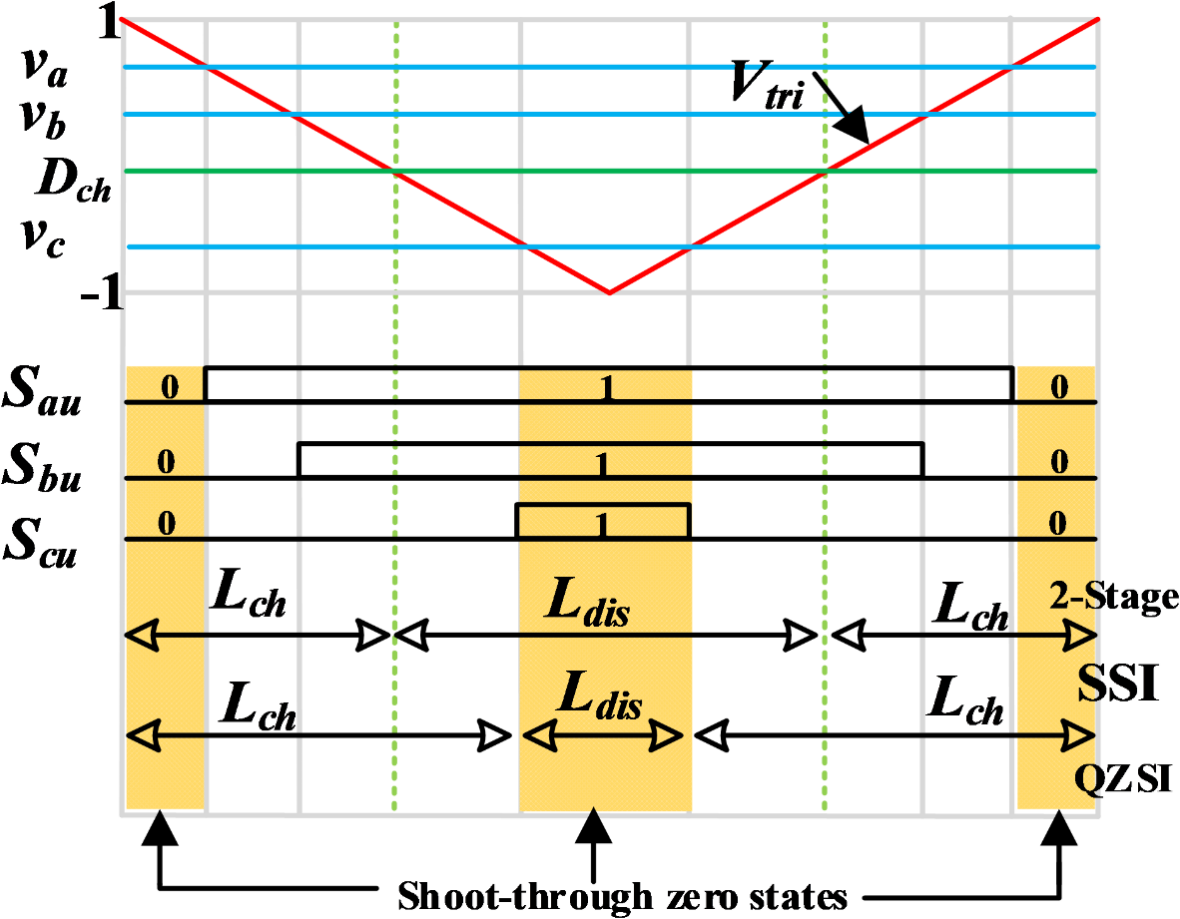
Comparison of the analysis modulations-Zoom on the reference and carrier signals for one switching cycle.

The modulation of the two-stage dc–dc–ac architecture can be divided into two separate modulators to perform the boosting and inversion actions, as shown in Fig. 3. The dc-boost unit is controlled by comparing the duty cycle ${D}_{ch}$ with the carrier wave, whereas the B6 switches are controlled via MSVM signals (${v}_{a}, {v}_{b}$, ${v}_{c}$)^10^. On the other hand, the q-ZSI is modulated as given by Abdelhakim et al. in^30^. The operation of the q-ZSI topology requires ST pulses to specify the output voltage^13^. In contrast, the operation of the SSI does not require any generation of special pulses or modifications of the conventional three-phase inverter (i.e., the VSI) for its standard operation. Hence, the same modulation scheme can be applied to the VSI and SSI. In this study, the MSVM presented by Abdelhakim et al.^9^ was utilized to obtain lower ripples in both the input current and the dc-link voltage and provide better output voltage waveforms. It is worth noting that dead-time zones must implement the SSI and the two-stage architectures, while in the q-ZSI, the dead-time is unnecessary.

#### Space vector PWM techniques

The space-vector $\overrightarrow{V}$ for each of the eight switching states of the standard VSI can be synthesized by:

1 $$\vec{V} = 2/3\left( { V_{an} + V_{bn} e^{j2\pi /3} + V_{cn} e^{j4\pi /3} } \right)$$

**Fig. 4:**
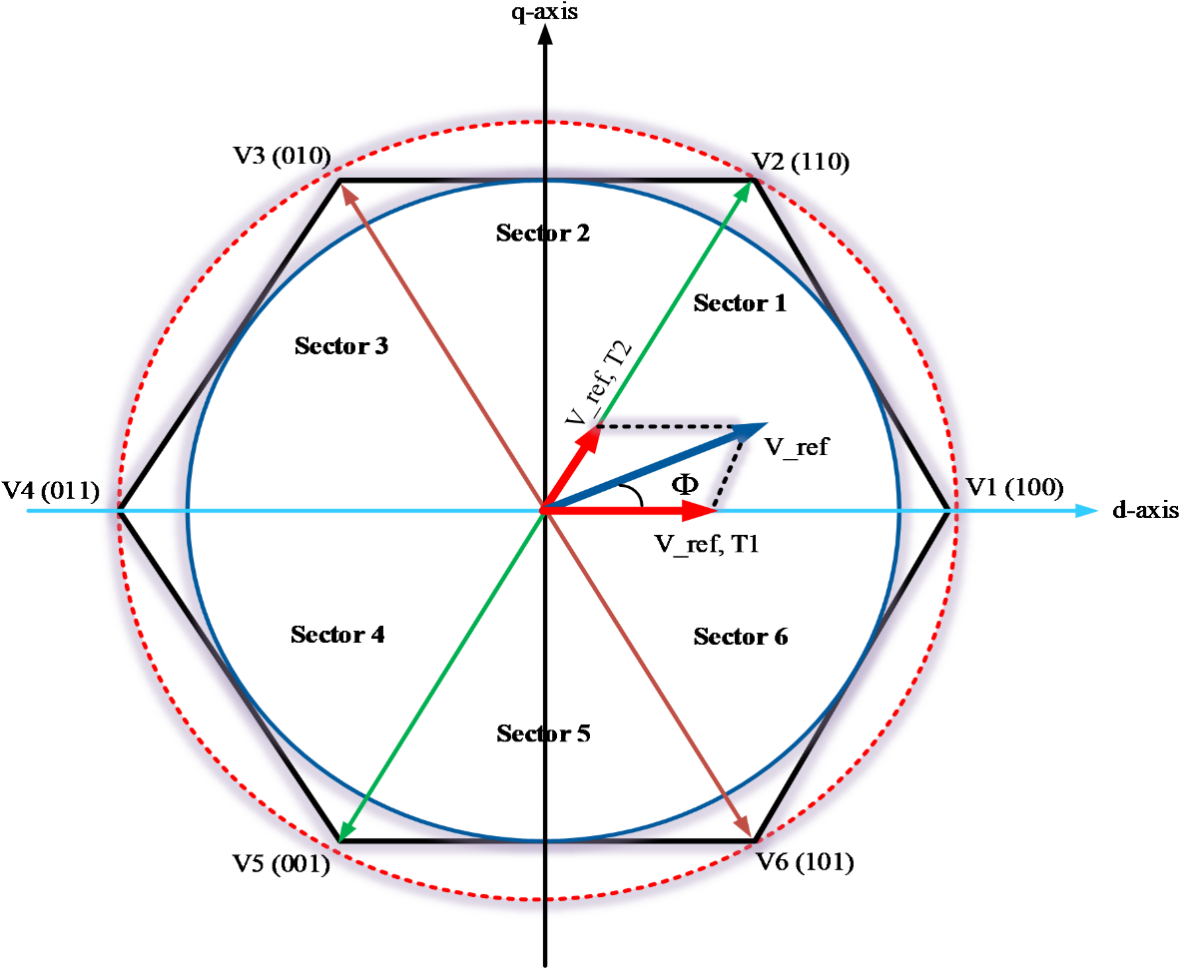
Switching vectors and sectors of traditional SVM used in the VSI.

Utilizing space vector modulation for the phase voltages of the inverter yields eight distinct voltage vectors, comprising six active vectors and two zero vectors. The resulting vectors, along with their corresponding instantaneous common mode voltage (CMV) magnitudes for each switching vector, are illustrated in Fig. 4. This representation is crucial for understanding how the different voltage vectors contribute to the overall performance of the inverter, particularly in terms of controlling output voltage and minimizing common mode voltage effects.

**Fig. 5:**
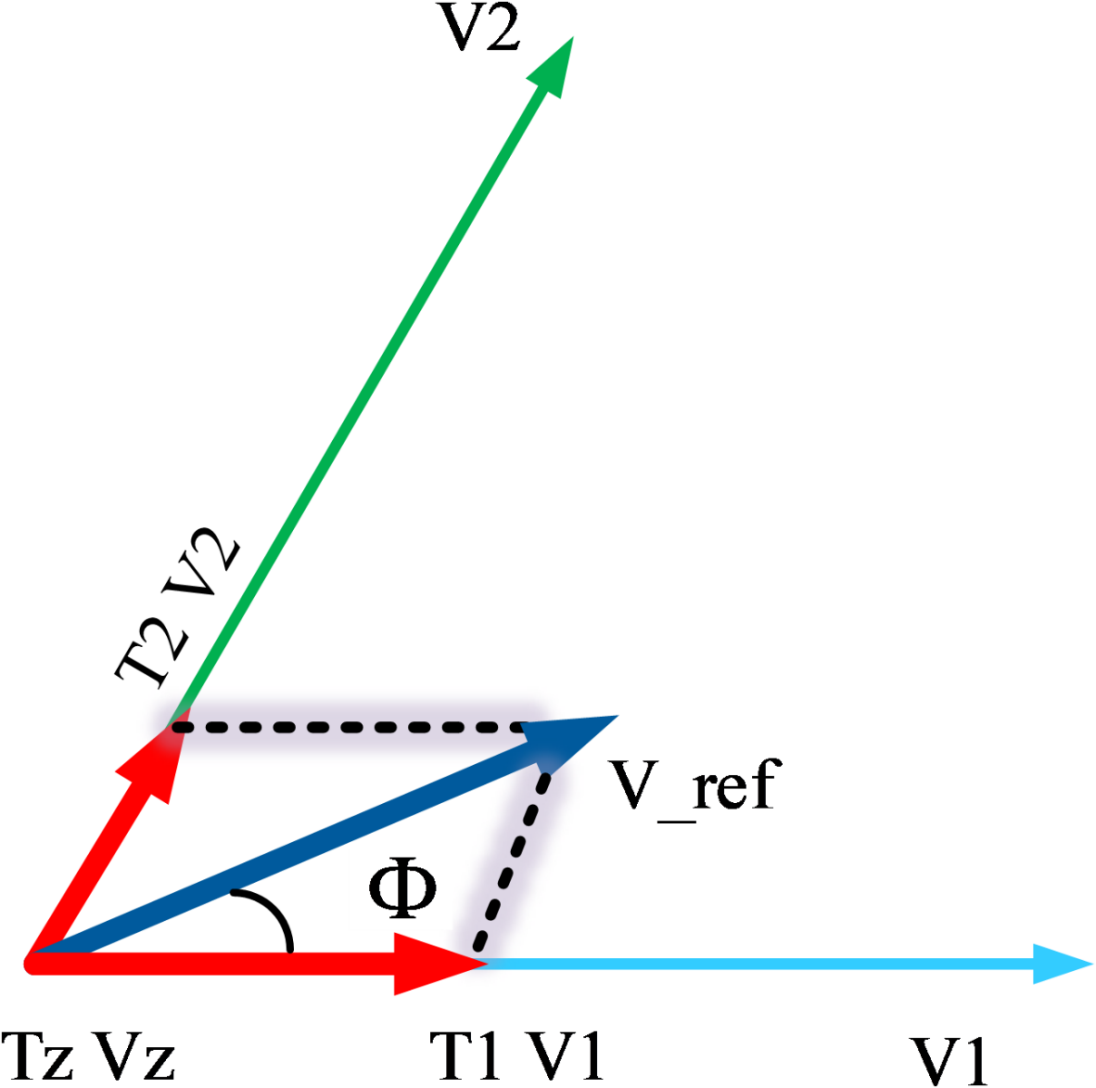
The generation of the reference vector for conventional SVPWM.

In conventional Space Vector Pulse Width Modulation (SVPWM) for a three-phase Voltage Source Inverter (VSI), the reference voltage vector $\left( {\vec{V}^{*} = {\text{Me}}^{{{\text{j}}\vartheta }} } \right)$ is synthesized using two adjacent active space vectors within each sector, along with zero vectors. For instance, if ${\overrightarrow{V}}^{*}$ falls within sector A1, the active vectors $\overrightarrow{{V}_{1}}$ and $\overrightarrow{{V}_{2}}$, as depicted in Fig. 5, are employed. The reference vector for each sample interval ${T}_{s}$ is then determined based on the volt-second balance principle. This approach ensures that the synthesized output voltage accurately matches the desired reference voltage over each sampling period, facilitating precise control of the inverter’s output while minimizing harmonic distortion.

2 $${{T}_{s}\overrightarrow{V}}^{*}={T}_{1}\overrightarrow{{V}_{1}}+{T}_{2}\overrightarrow{{V}_{2}}+{T}_{z}\overrightarrow{{V}_{z}}$$

where ${T}_{1},{T}_{2},{T}_{z}$ are the time of application of the active and zero vectors ${V}_{1},{V}_{2},{V}_{z}$ respectively, which are calculated by:

3 $$\begin{gathered} T_{1} = T_{s} M\sin (\pi /3 - \vartheta ), \hfill \\ T_{2} = T_{s} M\sin (\vartheta ), \hfill \\ T_{z} = T_{s} - T_{1} - T_{2} \hfill \\ \end{gathered}$$

In the conventional SVPWM, ${T}_{z}$ is equally shared between the zero vectors ${V}_{0}$ and ${V}_{7}$ with ${T}_{0}$ and ${T}_{7}$. Therefore,

4 $${T}_{0}={T}_{7}=.5{T}_{z}=(1-M\text{sin} (\pi /3-\vartheta ))$$

Equation (4) is valid for the entire linear region in the range of $0\le M\le 1.$

#### Operation of SSI

**Fig. 6:**
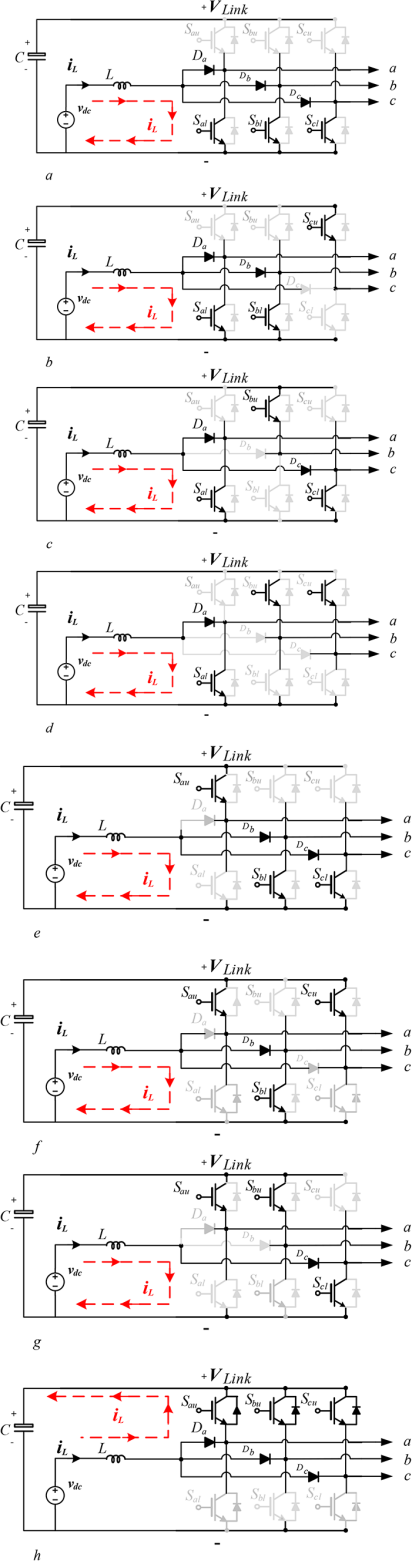
The eight operating states of a single-stage DC-AC split-source inverter.

The three-phase SSI uses the same B6-bridge as the conventional three-phase VSI, considering the same eight states as shown in Fig. 6.

This inverter uses at least one of the lower semiconductor switches ${S}_{al},{S}_{bl}$, and ${S}_{cl}$, to be ON to charge the inductor L, where seven different states exist. Meanwhile, it uses only one state to discharge this inductor and charge the inverter dc-link capacitor, as shown in Fig. 6h when all the upper switches ${S}_{au},{S}_{bu}$, and ${S}_{cu}$, are ON i.e., state 111. The charging of L can be done considering three semiconductor Switches as shown in Fig. 6a, or two semiconductor switches as shown in Fig. 6b, c, and e, or one semiconductor switch as shown in Fig. 6d, f, and g.

### Voltage gain and boosting action control

#### Ideal case

This section focuses on the voltage supplied to the quasi-Z-source inverter (q-ZSI) with a continuous input current. The inverter operates in two modes: shoot-through (ST) and non-shoot-through (non-ST) states. Accordingly, the following relationships are established^31^:

5 $$\left\{\begin{array}{c}{V}_{C1}=\frac{1-\delta }{1-2\delta }*E \\ {V}_{C2}=\frac{\delta }{1-2\delta }*E\end{array}.\right.$$

The peak dc-link voltage at the input of the inverter bridge can be expressed as

6 $${V}_{inv}=\frac{1}{1-2\delta }E=B*\text{E}$$

a Non-ST Mode

In this mode, the diode conducts forward, and the inverter bridge operates in six active states (V_1_ to V_6_) or two null states (V_0_ and V_7_). During this operation, the capacitors charge while the inductors transfer their stored energy to the load.

b ST Mode

This mode occurs when the Z-network is short-circuited by any inverter leg. In this state, the combined voltage across the two capacitors exceeds the supply voltage $\text{E}$, causing the diode to become reverse-biased. The capacitors then charge the inductors, while the DC-link voltage ${V}_{inv}$ becomes zero.

In contrast, during the non-ST mode, the peak value of the DC-link voltage ${V}_{inv}$ can be expressed as shown in Eq. (6).

where B denotes the boost factor of the q-ZSI. Utilizing the concept of power invariance, the average inductive currents flowing through ${L}_{1}$ and ${L}_{2}$ can then be calculated. This analysis is essential for understanding the voltage boosting capabilities of the q-ZSI and for ensuring efficient power conversion in various applications . By comprehensively evaluating these relationships, engineers can optimize the design and performance of the inverter, ultimately enhancing its operational efficiency .

7 $${I}_{L1}={I}_{L2}={I}_{in}=P/{V}_{dc}$$

The SSI, however, has two operational modes:

i. L-charging mode:

8 $$\left\{\begin{array}{l}E=r {i}_{1}+L\frac{\partial li}{\partial t}\\ {V}_{L}=E-r {i}_{1}\end{array}\right.$$

ii. L-discharging mode:

9 $$E={V}_{L}+{V}_{C}+ r {i}_{2},$$

10 $${V}_{dc}={V}_{C}=E-{V}_{L}- r {i}_{2}.$$

The ideal case can be analyzed by setting $\frac{\partial }{\partial t}$ ${V}_{L}= 0$ as

11 $$E {T}_{1}=-\left(E-{V}_{C}\right){T}_{2}, {V}_{C}{T}_{2}=E({T}_{2}+{T}_{1})$$

where ${V}_{C}=E/D$ and $D=(1-M)$, and M is the modulation index.

#### Non-ideal case (Considering the inductor resistance)

A non-ideal case can be analyzed by setting $\frac{\partial }{\partial t}{V}_{L}\ne 0$.

12 $${V}_{L}=E-r {i}_{L}$$

13 $$E {T}_{1}-r {i}_{L} {T}_{1}=-E {T}_{2}+{V}_{C} {T}_{2}+r {i}_{L} {T}_{2}$$

14 $${V}_{C}=\frac{E}{D}-\frac{r }{D} \frac{\partial }{\partial t}{i}_{L}$$

The inductive current ${I}_{L}$ can be calculated using the power balance equation as

15 $${P}_{o}={P}_{in}-{P}_{r}$$

16 $$\frac{3}{2} {\widehat{V}}_{ph} {\widehat{I}}_{ph} \mathit{cos}\phi =E\frac{\partial }{\partial t} {I}_{L}-\frac{\partial }{\partial t}{{I}_{L}}^{2}r$$

where ${i}_{L}$ represents average inductive current.

17 $${I}_{L}=\frac{E\pm \sqrt{{E}^{2}-4r\frac{3}{2} {\widehat{V}}_{ph} {\widehat{I}}_{ph} \mathit{cos}\phi }}{2r}$$

18 $${V}_{C}=\frac{B E }{2}\left\{1+\sqrt{1- \frac{2r }{Z} {G}^{2}\mathit{cos}\phi } \right\}$$

19 $${\widehat{V}}_{ph} r=\frac{M }{\sqrt{3}}\frac{B E }{2}\left\{1+\sqrt{1- \frac{2r }{Z} {G}^{2}\mathit{cos}\phi } \right\}$$

20 $${G}_{r}=\frac{M }{2(1-M)}\left\{1+\sqrt{1- \frac{2r }{Z} {G}^{2}\mathit{cos}\phi \frac{{M}^{2} }{(1-{M)}^{2}}} \right\}$$

The modulation index of an SSI can be calculated as

21 $$M=\left(3Z+2\sqrt{6 } \sqrt{Z*r*\mathit{cos}\phi }\right)/\left(3Z*8r*\mathit{cos}\phi \right),$$

where

22 $$\left\{\begin{array}{l}{\widehat{V}}_{ph} =\frac{M B }{\sqrt{3}} E=\frac{G }{\sqrt{3}} E, \\ {\widehat{V}}_{ph} r=\frac{G r}{\sqrt{3}} E\end{array}\right..$$

## Mathematical derivation

The SB-MSVM strategy proposed for the three-phase q-Z-source inverter (q-ZSI) was mathematically formulated based on the methodologies outlined by Peng^26^ and Zdanowski et al.^13^. This formulation involved the approach introduced by Abdelhakim et al.^30^, which facilitated the estimation of the necessary inductance and capacitance values for the system. The expressions derived for these parameters are as follows, the inductance ${L}_{q}$ can be calculated using the formula:

23 $${L}_{q}=\frac{M\cdot (1-M)\cdot E}{\left(2M-1\right) \cdot 2{f}_{s} \cdot \Delta I},$$

where $M$ represents the modulation index, $E$ is the input voltage, ${f}_{s}$ denotes the switching frequency, and $\Delta I$ signifies the peak-to-peak ripple in the inductor current. This relationship highlights the interplay between the modulation index and the inductance value, emphasizing how varying these parameters affects system performance, The capacitance ${C}_{1}$ required for the first capacitor can be derived from the following equation:

24 $${C}_{1}=\frac{(1-M)\cdot {I}_{in}}{{f}_{S}\cdot \Delta {Vc}_{1}},$$

Here, ${I}_{in}$ represents the average input current, and $\Delta {Vc}_{1}$ is the peak-to-peak voltage ripple across the capacitor ${C}_{1}$. This equation underscores the significance of the modulation index and input current in determining the capacitance needed to maintain voltage stability, Similarly, for the second capacitor, the capacitance ${C}_{2}$ can be expressed as:

25 $${C}_{2}=\frac{(1-M)\cdot {I}_{in}}{{f}_{S}\cdot \Delta {Vc}_{2}},$$

In this case, $\Delta {Vc}_{2}$ indicates the peak-to-peak voltage ripple across capacitor ${C}_{2}$. This equation further demonstrates the role of the modulation index and input current in establishing the appropriate capacitance levels necessary for effective operation.

By employing the Simple Boost Maximum Space Vector Modulation (SB-MSVM) strategy, the duty cycle ${D}_{o}$ can be decoupled from the modulation index MMM, allowing for the introduction of two control parameters suitable for closed-loop applications based on the mathematical derivation of the Split-Source Inverter (SSI). The required capacitance and inductance values can be determined similarly to those in a boost converter by focusing solely on the ripple associated with the switching frequency^10^. This approach facilitates precise control over the system’s dynamic response, ensuring stability and optimal performance in various operating conditions.

26 $${L}_{s}=\frac{{M}_{s}\cdot E}{{f}_{s} \cdot \Delta I},$$

27 $${C}_{s}=\frac{(1-{M}_{s})\cdot {I}_{in}}{{f}_{s} \cdot \Delta {V}_{dc}},$$

where the mathematical derivation of the two-stage boost inverter employs the same modulation strategy. Following the approach introduced by^32,33^, the required inductance was estimated as

28 $${L}_{two}=\frac{E\cdot D}{{f}_{s} \cdot \Delta I}$$

From Eqs. (23) and (26),

29 $${L}_{qZSI}/{L}_{SSI}=\left\{\frac{{M}_{Z}\cdot (1-{M}_{Z})\cdot E}{\left(2{M}_{Z}-1\right) \cdot 2{f}_{s} \cdot \Delta I}\right\}/\left\{\frac{{M}_{S}\cdot E}{{f}_{s} \cdot \Delta I}\right\}$$

30 $${L}_{qZSI}/{L}_{SSI}=\frac{{M}_{Z}\cdot (1-{M}_{Z})}{2\cdot \left(2{M}_{Z}-1\right) \cdot {M}_{S}}.$$

From Eqs. (26) and (28),

31 $${L}_{TWO}/{L}_{SSI}=\left\{\frac{E\cdot D}{{f}_{s} \cdot \Delta I}\right\}/\left\{\frac{{M}_{S}\cdot E}{{f}_{s} \cdot \Delta I}\right\},$$

32 $${L}_{TWO}/{L}_{SSI}=\frac{{D}_{TWO}}{{M}_{s} }.$$

To obtain the modulation indexes for q-ZSI, SSI, and the two-stage boost inverter for the same gain. The normalized fundamental output peak phase voltage (${\widehat{V}}_{ph}/E$) for q-ZSI can be estimated by

33 $${G}_{qZSI}=\frac{{\widehat{V}}_{ph}}{E}=\frac{M}{\sqrt{3} (2M-1)}.$$

The SSI can then be estimated by

34 $${G}_{SSI}=\frac{{\widehat{V}}_{ph}}{E}=\frac{M}{\sqrt{3} (1-M)}.$$

The two-stage can be estimated by

35 $${G}_{TWO}=\frac{{\widehat{V}}_{ph}}{E}=\frac{M}{\sqrt{3} (1-{D}_{ch})}.$$

For the same voltage gain in the output. From Eq. (33),

36 $${M}_{qZSI}=\frac{\sqrt{3} \cdot {G}_{qZSI}}{2\sqrt{3} (G-1)}.$$

From Eq. (34),

37 $${M}_{SSI}=\frac{\sqrt{3} \cdot {G}_{SSI}}{\sqrt{3} G+1}.$$

From Eq. (35),

38 $${M}_{TWO}=\sqrt{3} \left(1-{D}_{ch}\right){ \cdot G}_{TWO}.$$

The advantages gained from using the MSVPWM strategy rather than traditional continuous modulation strategies are summarized in Table 1. The MSVPWM strategies achieved the fewest commutations using the fewest reference signals. Employing the SV-MSVM strategy allowed for the following advantages:

- The effective switching frequency of the switches (${S}_{au},{S}_{bu},\text{ and }{S}_{cu}$) is equal to two-thirds of the carrier frequency.
- The effective switching frequency of the switches (${S}_{al},{S}_{bl}, \text{and} {S}_{cl}$) is equal to the carrier frequency.
- The number of switching variations was reduced from traditional modulation strategies.
- Relieve one every time.
- Simple generating gate signals.

**Table 1:** The parameters of the prototypes studied are summarized in this table.

Inverter features	Two-stage	q-ZSI^27^	SSI
Circuit diagram	Figure 1a	Figure 1b	Figure 1c
No. of switches	7	6	6
No. of diodes	1	1	3
No. of inductors	1	2	1
No. of capacitors	1	2	1
THD of the output voltage	Lowest	Low at low-voltage gains	Low at high voltage gains
Modulation Strategy	SBMSV	SBMSV	SBMSV
Reliability	Low	Low	Low
PWM Complexity	Moderate	Complex	Simple
Input current waveform	Continuous	Continuous	Continuous
Boosting factor	$\frac{1}{1-{D}_{ch}}$	$\frac{1}{1-{2D}_{sh}}$	$\frac{1}{{\delta }_{l}}$
Voltage gain	$\frac{M}{\sqrt{3} (1-{D}_{ch})}$	$\frac{M}{\sqrt{3} (2M-1)}$	$\frac{M}{\sqrt{3} (1-M)}$
Capacitor voltage	$\frac{1}{(1-{D}_{Ch})}$	$\frac{{V}_{C2}}{E}=\frac{{D}_{Sh}}{(1-{2D}_{Sh})}$	$\frac{1}{(1-{D}_{Ch})}$
Voltage stresses	Lowest	Highest	Low
Voltage gain control	Simple	Complex	Moderate
dc-voltage, *V*_*dc*_	Continuous	Pulsed	Continuous
Efficiency	88%	89%	93%

**Fig. 7:**
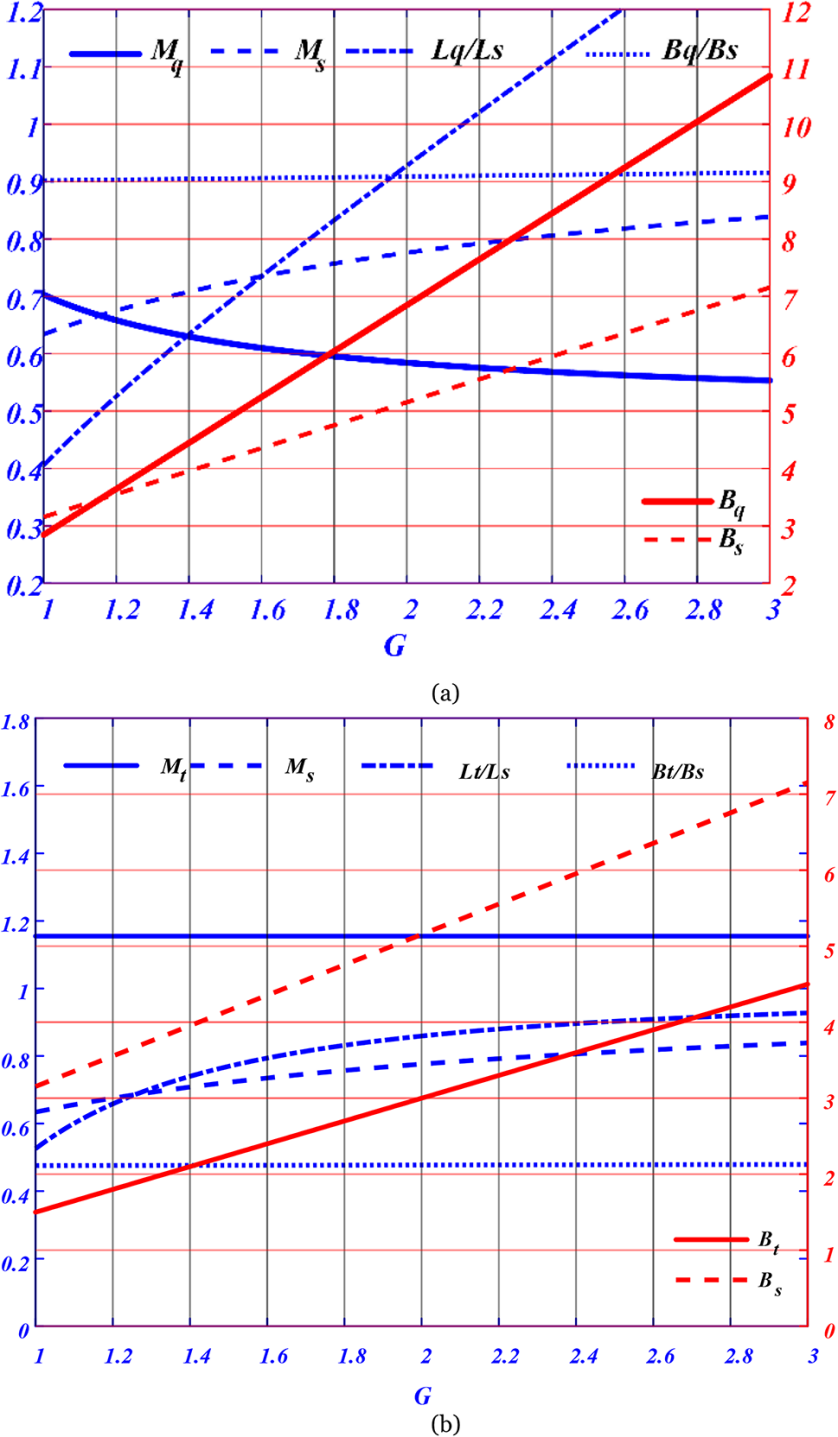
outcomes of analytical comparisons between the various topologies examined. (**a**) Results of the Split-Source Inverter (SSI) in relation to the quasi-Z-source inverter (q-ZSI), while (**b**) comparison between the SSI and the two-stage topology.

The analyzed topologies are compared in Fig. 7, where the SSI topology is compared with that of the q-ZSI topology in Fig. 7a and that of the two-stage topology in Fig. 7b.

The implementation of the modulation technique relies on the use of comparators and the quantity of modulating signals. Among the options analyzed, the Split-Source Inverter (SSI) is the simplest to modulate, as it requires only the duty cycles for each phase. In contrast, both the standard DC–DC–AC converter and the quasi-Z-source inverter (q-ZSI) necessitate a greater number of modulating signals and comparators.

However, the DC–DC–AC topology offers more flexibility in controlling voltage gain compared to the q-ZSI topology, as the inverter’s operation does not restrict the performance of the boosting stage. Overall, the proposed Simple Boost Maximum Space Vector Modulation (SB-MSVM) strategy effectively achieves the same voltage gain and operational stresses as the Simple Boost Space Vector (SBSV) method while incorporating the boost actions represented by (${B}_{S},{B}_{q}$), Additionally, it balances the inductances $({L}_{qZSI}/{L}_{SSI})$ and the modulation indexes (${M}_{qZSI},{M}_{SSI}$) between the SSI and the q-ZSI. This versatility positions the SB-MSVM strategy as a promising approach for enhancing the performance of inverter systems in various applications.

## Simulation and experimental validation

### Simulation validation

To further analyze the three topologies, they were simulated via MATLAB/SIMULINK^®^; the parameters used are listed in Table 2.

**Table 2:** Parameters used for the simulation of the analyzed topologies.

Parameter	Two-stage	q-ZSI	SSI
*M*1	$\frac{\sqrt{3} }{2}$	0.612	0.838
Frequency ${F}_{I}$	20,000 HZ		
Frequency ${F}_{O}$	50 HZ		
Inductance and capacitance	*L* = 5 mH	*L* = 5 mH	*L* = 5 mH
	*C* = 470 μF	*C* = 470 μF	*C* = 470 μF
dc voltage	100 V		
Boosting factor	3		

The analyzed topologies of the three-phase inverters were configured to supply a three-phase inductive load (10-Ω resistance in series with 5-mH inductance) from a low-voltage dc supply; an input dc voltage or Photovoltaic Panel of 100 V was assumed for the simulation, whereas 20 V was used in the experimental design. The required load power and voltage, according to the site temperature and solar conditions and the possible boosting gain range, we can estimate the maximum power voltage range. Using any of the known MPPT method [perturb and observe, incremental conductance, etc], the DC gain can be altered to reach the maximum point, and at the same time, the required output voltage is secured by the suitable modulation index. A leading-edge sawtooth carrier wave with a frequency of 20 kHz was used. For the SSI, the inductance and capacitance of the dc-link capacitor were assumed as 5 mH and 470 µF. For the q-ZSI, the network impedance and capacitance were assumed as 5 mH and 470 μF, whereas those of the two-stage dc–dc–ac converter were 5 mH and 470 μF.

**Fig. 8:**
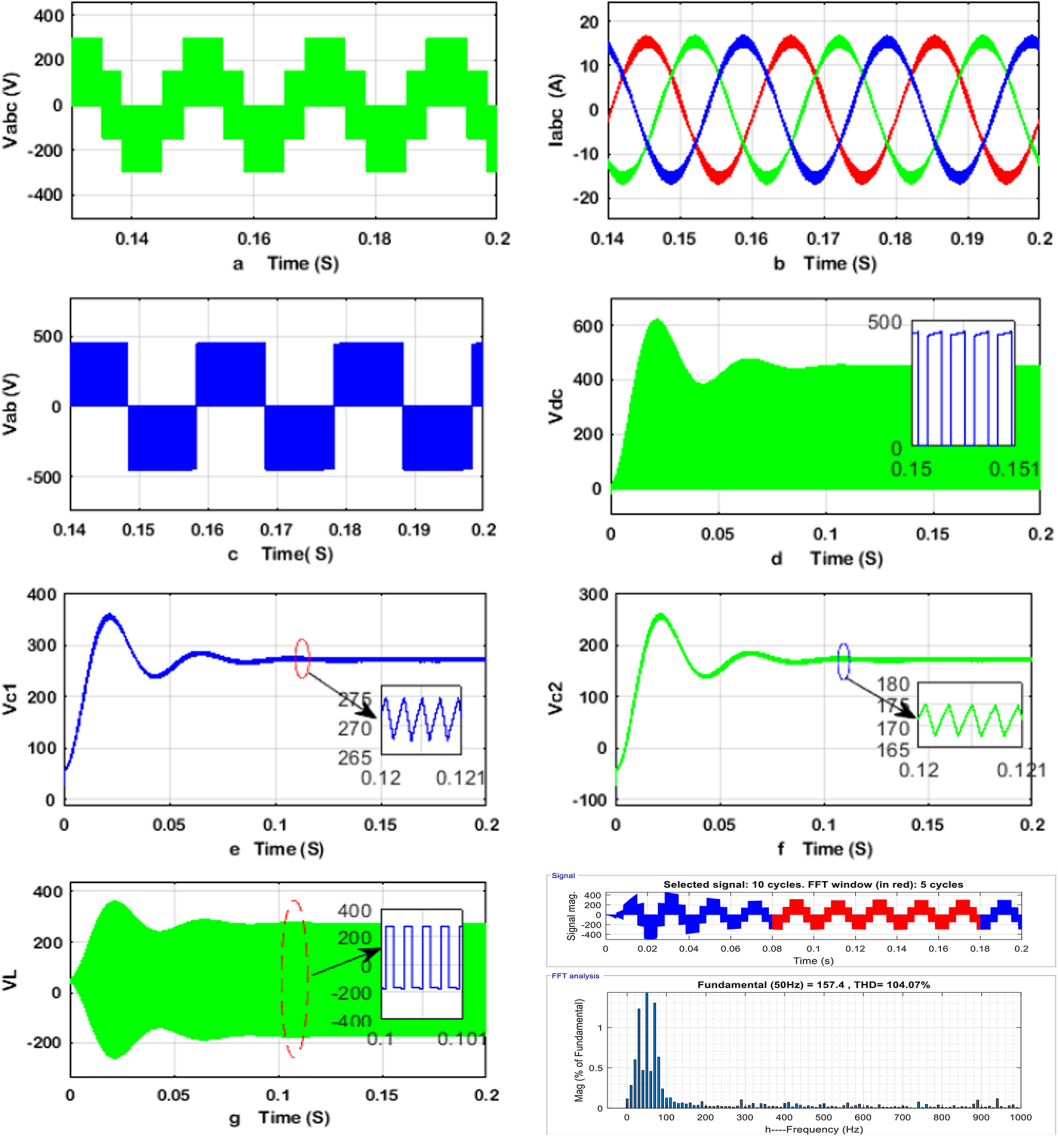
Simulation results of the q-ZSI using the proposed SB-MSVM strategy, where (**a**) inverter phase voltage, (**b**) output line currents, (**c**) inverter line voltage, (**d**) dc-link voltage, voltage of the (**e**) C1 and (**f**) C2 capacitors, (**g**) inductor voltage waveforms, and (**h**) THD analysis of q-ZSI inverter at phase voltage.

**Fig. 9:**
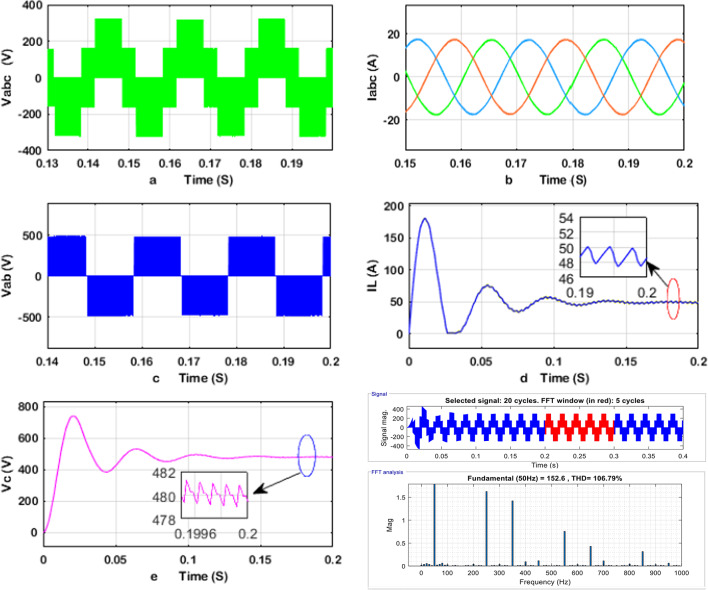
Simulation results of the SSI using the proposed SB-MSVM strategy, where (**a**) inverter phase voltage, (**b**) output line currents, (**c**) inverter line voltage, (**d**) inductor current, (**e**) capacitor voltage waveforms, and (**f**) THD analysis of SSI inverter at phase voltage.

**Fig. 10:**
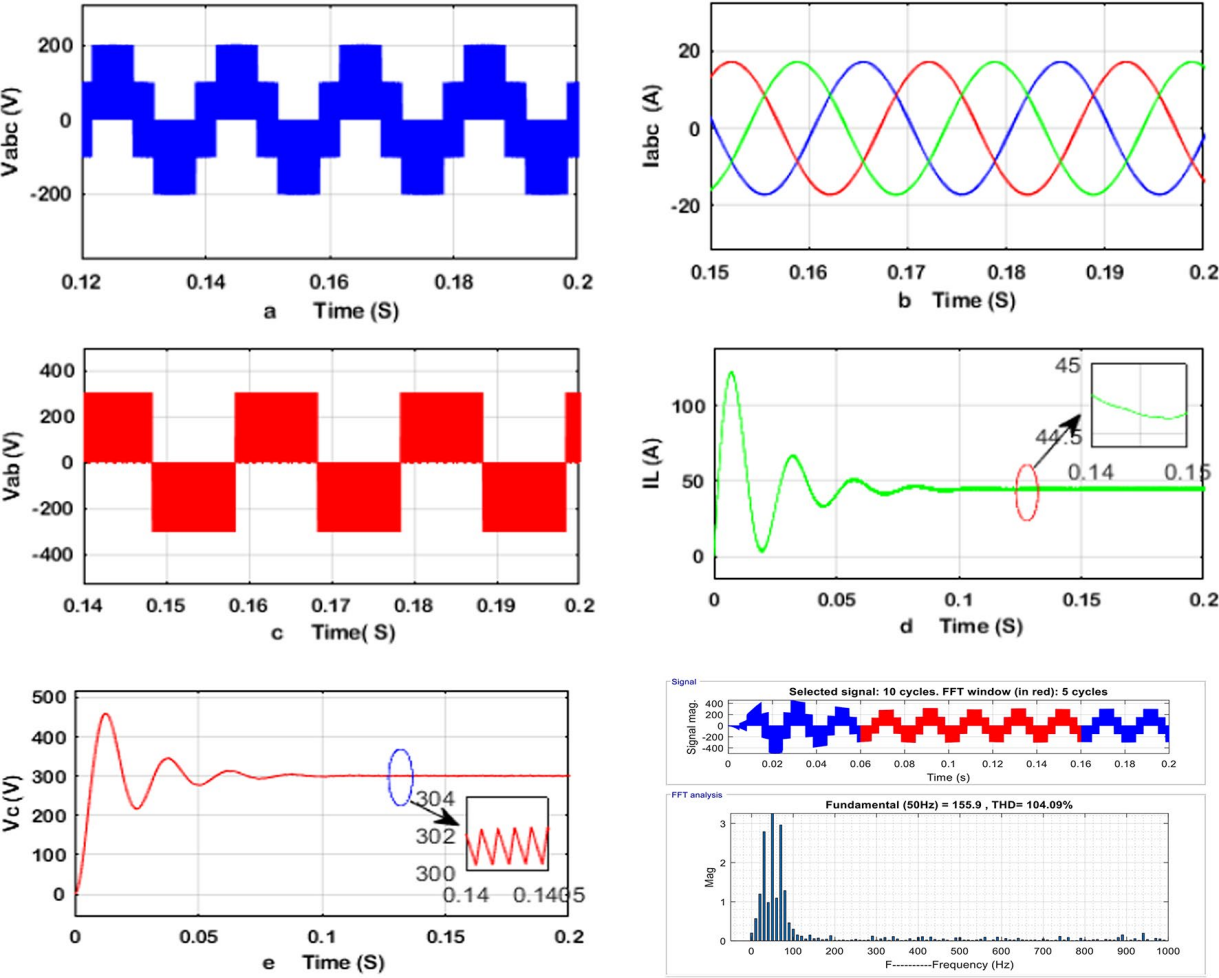
Simulation results of the traditional two-stage inverter using the proposed MSVM strategy, where (**a**) inverter phase voltage, (**b**) output line currents, (**c**) inverter line voltage, (**d**) inductor current and (**e**) capacitor voltage waveforms, and (**f**) THD analysis of traditional two-stage inverter at phase voltage.

The obtained simulation results of the q-ZSI, SSI, and two-stage three-phase inverter are shown in Figs. 8, 9, and 10, including the phase and line voltages, output currents, and capacitor voltage, dc-link voltage waveforms and the total harmonic distortion analysis of inverters at phase voltage. The simulation results exhibit high-quality sinusoidal output currents. The detailed simulation results for the q-ZSI, SSI, and two-stage three-phase inverter are presented in Figs. 8, 9, and 10, highlighting critical performance parameters such as phase and line voltages, output currents, capacitor voltages, DC-link voltage waveforms, and total harmonic distortion (THD) analysis at the phase voltage.

Upon examination, the simulation results reveal that all three inverter configurations successfully generate high-quality sinusoidal output currents, which are essential for maintaining grid compatibility and load efficiency. The phase voltage waveforms are smooth and free from significant distortions, indicating effective modulation strategies for stable power delivery.

The THD analysis shows notable differences between the configurations. The SSI demonstrates superior harmonic performance, with lower THD compared to the two-stage DC–DC–AC inverter, thanks to its continuous current path and shoot-through capability that enhances power quality. The q-ZSI also performs well due to its inherent capability to boost and condition the voltage in a single stage, thereby reducing switching losses and harmonic content.

Capacitor and DC-link voltage waveforms provide insight into the stability of energy storage and transfer within each topology. The SSI maintains a more stable capacitor voltage, contributing to smoother inverter operation and enhanced power conversion efficiency. Meanwhile, the two-stage configuration shows larger voltage fluctuations, which could affect long-term reliability and thermal management.

These findings underscore the advantages of single-stage inverter topologies, particularly the SSI and q-ZSI, in terms of reduced harmonic distortion, improved energy management, and enhanced output quality. The comparative simulation analysis highlights the potential of these advanced inverter designs for transformerless photovoltaic systems and other renewable energy applications.

### Experimental validation

**Fig. 11:**
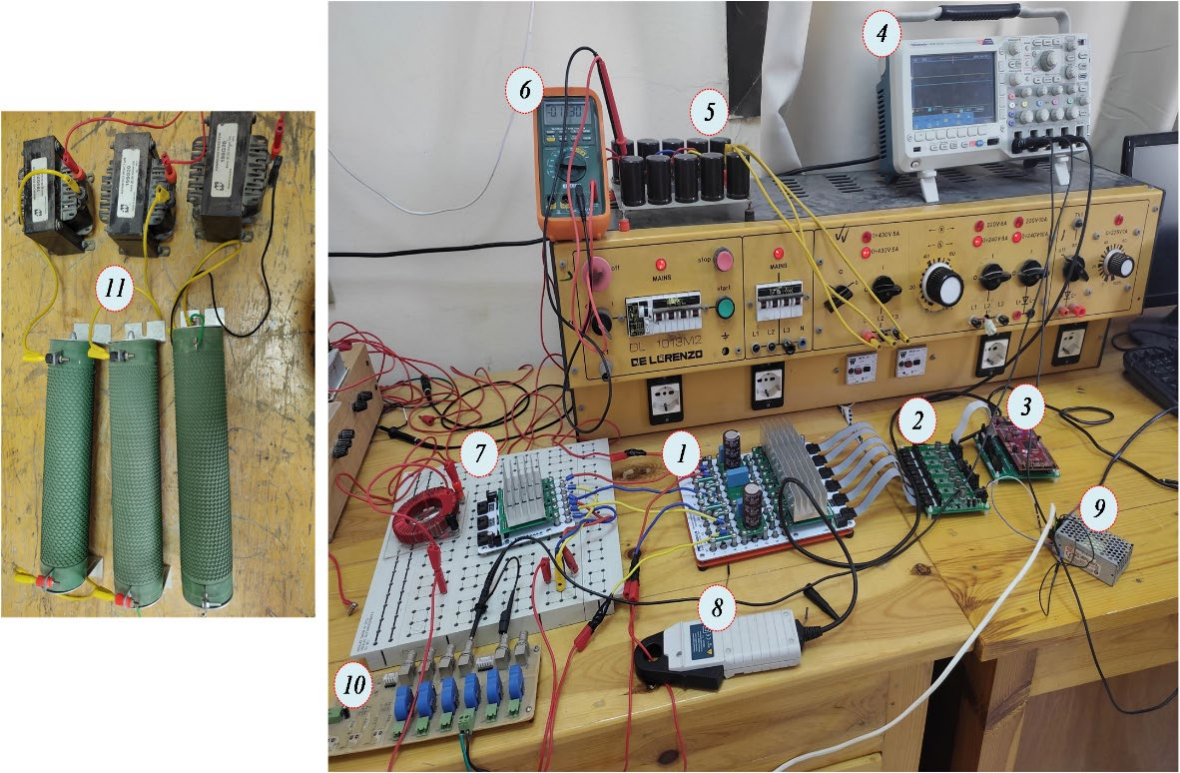
Photographs of the experimental setup, where (1) six IGBT modules, (2) Six Modules Signal Collector Board, (3) F28379D Launchpad Kit Card™, (4) The oscilloscope, (5) The rectifier circuit, (6) digital multimeter, (7) ${L}_{S}$ With three fast-recovery diodes, (8) Clamp ampere, (9) dc supply, (10) Six current sensors and (11) RL-loads.

To verify the performance of the analyzed topologies, a small-scale prototype of the Split-Source Inverter (SSI) was developed and implemented in the laboratory, utilizing a custom-designed and configurable power electronics development kit from PEModule^®^^42^. The parameters employed in the prototype are detailed in Table 3, and the experimental setup is depicted in Fig. 11. The SSI was constructed using the Texas Instruments LaunchPad LAUNCHXL-F28379D DSP board, which is responsible for generating the gating pulses for the Maximum Space Vector Modulation (MSVM) and the associated dead-time generation, set at 2 µs. Based on equations (26) and (27), inductor and capacitor values of 2.5 mH and 150 µF were selected for this implementation. This experimental setup allows for comprehensive testing and analysis of the SSI’s performance, enabling further insights into its operational characteristics and potential applications in power conversion systems

**Table 3:** Experimental study parameters for the SSI.

Parameter	Value	Parameter	Value
Switching frequency	20 kHz	DSP	TI-F28379D
Dead time	2 µs	M	.5
Inductor	2.5 mH	Load	11 Ω & 5 mH
Capacitor	150 µF/400 V

**Fig. 12:**
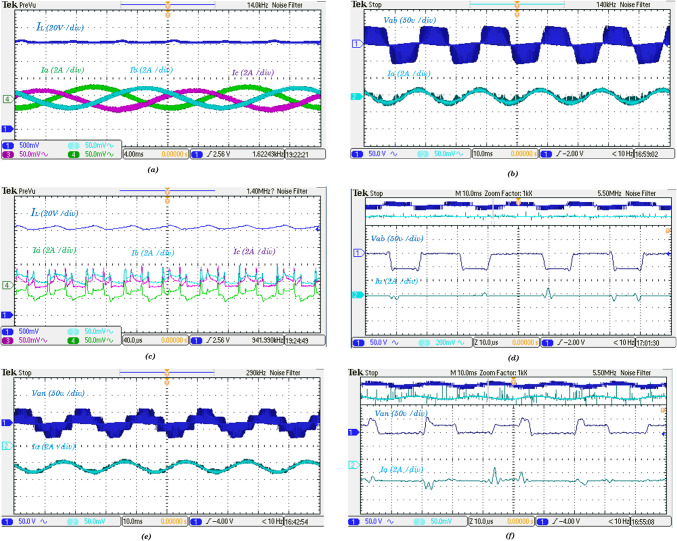
Obtained experimental results of the three-phase SSI using the proposed SB-MSVM strategy, where (**a**) dc current and output line currents (${I}_{a}$,${I}_{b,}{I}_{c}$), (**b**) line voltage, ${I}_{b}$, (**c**) zoomed-in views of dc current and output line currents (${I}_{a}$,${I}_{b,}{I}_{c}$), capacitor voltage, (**d**) zoomed-in views of line voltage, ${I}_{b}$, (**e**) phase voltage and output line current ${I}_{b}$, and (**f**) zoomed-in views of the phase voltage and output line current ${I}_{b}$ waveforms.

The TI-F28379D DSP board, highlighting its real-time control capabilities, high processing speed, and compatibility with advanced modulation strategies, the results of the case studies conducted on the Split-Source Inverter (SSI), detailed in Table 1, are illustrated in Fig. 12, which presents various waveforms that offer critical insights into the inverter’s performance. Panel (a) showcases the captured waveforms of the input and output currents, revealing how the SSI interacts with the input source and delivers power to the load. Panel (b) further depicts the output line voltage and output current ${I}_{b}$, along with the output phase voltage, highlighting the inverter’s ability to maintain voltage levels while supplying current. To provide a deeper understanding of the transient behavior, zoomed-in views of a one-millisecond interval for the input and output currents, as well as the DC-link voltage, are shown in panels (c), (d), and (f), respectively. These detailed views are essential for assessing current ripple, load regulation, and the stability of the DC-link voltage, which directly influences the quality of the AC output. Collectively, these waveforms facilitate a comprehensive analysis of the SSI’s operational efficiency and effectiveness, informing potential design optimizations and enhancing the overall performance of power electronic systems.

## Conclusion

This paper presents a comparative analysis of the three-phase Split-Source Inverter (SSI), quasi-Z-source inverter (q-ZSI), and the conventional two-stage DC–DC–AC inverter. The findings indicate that the SSI outperforms both the q-ZSI and the two-stage boost inverter. Furthermore, the SSI can utilize the same standard modulation techniques as the traditional two-stage DC–DC–AC inverter, while also accommodating continuous input current and offering a shorter commutation path compared to the q-ZSI. Additionally, the modified Space Vector Pulse Width Modulation (SVPWM) scheme significantly enhances the SSI’s performance by effectively mitigating low-frequency components in the input inductor current and DC-link capacitor voltage. This improvement underscores the advantages of adopting the Simple Boost Maximum Space Vector Modulation (SB-MSVM) strategy, which optimizes system efficiency and stability. The comparative assessment presented in this paper offers valuable insights for researchers, enabling a deeper understanding of the differences between various modulation schemes and guiding the selection of the most appropriate technique for specific power conversion applications.

Furthermore, this paper also introduces a comparative study between the analyzed topologies. These topologies (i.e., the conventional two-stage inverter, SSI, and q-ZSI) were theoretically analyzed and verified using MATLAB/SIMULINK^®^. The SSI experimental prototype was then investigated.

## Data Availability

The datasets generated during and/or analyzed during the current study are available from the corresponding author on reasonable request.

## References

[1] Wageh, M., Dabour, S. M., Mostafa, R. M. & Ghalib, M. A. Space vector PWM of three-phase inverter with MPPT for photovoltaic system. *Aust. J. Electr. Electron. Eng.* **18**(4), 310–318. 10.1080/1448837X.2021.1982445 (2021).

[2] Cossutta, P., Aguirre, M., Cao, A., Raffo, S. & Valla, M. Single-stage fuel cell to grid interface with multilevel current-source inverters. *IEEE Trans. Ind. Electron.* **62**(8), 5256–5264 (2015).

[3] Shehata, E. G., Thomas, J., Brisha, A. M., & Wageh, M. Design and analysis of a quasi y-source impedance network dc-dc converter. in *2017 Nineteenth International Middle East Power Systems Conference (MEPCON)* 235–241. 10.1109/MEPCON.2017.8301189. (IEEE, 2017).

[4] Bhowmick, S., Mukherjee, D., Basu, T. S. & Chakraborty, C. A three-phase four-leg neutral point clamped photovoltaic inverter with decoupled active & reactive power control and DC link voltage ripple minimization under unbalanced grid operation. in *IEEE Trans. Power Electron. *10.1109/TPEL.2025.3538166.

[5] Abdelhakim, A. Analysis and modulation of the buck-boost voltage source inverter (BBVSI) for lower voltage stresses. in *Proc. IEEE Int. Conf. Ind. Technol*. 926–934. (2015).

[6] Ahmed, H. F. & Siddiqui, D. Single-phase non-isolated inverter with shared-ground and broad input voltage operation. *IEEE J. Emerg. Sel. Top. Ind. Electron.*10.1109/JESTIE.2025.3538739 (2025).

[7] Diaz-Bustos, M., Baier, C. R., Torres, M. A., Melin, P. E. & Acuna, P. Application of a control scheme based on predictive and linear strategy for improved transient state and steady-state performance in a single-phase quasi-Z-source inverter. *Sensors* **22**(7), 2458 (2022). 35408073 10.3390/s22072458 PMC9003398

[8] Anand, V., Singh, V. & Mohamed Ali, J. S. Dual boost five-level switched-capacitor inverter with common ground. *IEEE Trans. Circuits Syst. II Express Briefs* **70**(2), 556–560. 10.1109/TCSII.2022.3169009 (2023).

[9] Abdelhakim, A., Davari, P., Blaabjerg, F. & Mattavelli, P. An improved modulation strategy for the three-phase Z-source inverters (ZSIs). in *IEEE Energy Conv. Cong. and Expo. (ECCE)* 4237 (2017).

[10] Abdelhakim, A., Mattavelli, P. & Spiazzi, G. Three-phase split- source inverter (SSI): Analysis and modulation. *IEEE Trans. Power Electron.* **31**(11), 7451–7461 (2016).

[11] Dabour, S. M., Abdel-Khalik, A. S., Ahmed, S. & Massoud, A. M. A family of discontinuous PWM strategies for quasi Z-source nine-switch inverters. *IEEE Access* **9**, 169161–169176 (2021).

[12] Jakhar, A. & Sandeep, N. Switched-capacitor-based seven-level boosting inverter with reduced voltage stress for grid-connected photovoltaic applications. *IEEE J. Emerg. Sel. Top. Ind. Electron.*10.1109/JESTIE.2025.3535917 (2025).

[13] Zdanowski, M., Peftitsis, D., Piasecki, S. & Rabkowski, J. On the design process of a 6-kva quasi-z-inverter employing sic power devices. *IEEE Trans. Power Electron.* **31**(11), 7499–7508 (2016).

[14] Battiston, A., Miliani, E. H., Pierfederici, S. & Meibody-Tabar, F. A novel quasi-z-source inverter topology with special coupled inductors for input current ripples cancellation. *IEEE Trans. Power Electron.* **31**(3), 2409–2416 (2016).

[15] Liu, Y., Abu-Rub, H. & Ge, B. Z-source/quasi-z-source inverters: Derived networks, modulations, controls, and emerging applications to photovoltaic conversion. *IEEE Ind. Electron. Mag.* **8**(4), 32–44 (2014).

[16] Liu, J., Wu, J., Qiu, J. & Zeng, J. Switched Z-source/quasi-Z-source DC-DC converters with reduced passive components for photovoltaic systems. *IEEE Access* **7**, 40893–40903 (2019).

[17] Ge, B. et al. An energy-stored quasi-Z-source inverter for application to photovoltaic power system. *IEEE Trans. Ind. Electron.* **60**(10), 4468–4481 (2012).

[18] Jamal, I. et al. A comprehensive review of grid-connected PV systems based on impedance source inverter. *IEEE Access* **10**, 89101–89123 (2022).

[19] Abu-Zaher, M., Zhuo, F., Orabi, M., Hassan, A. & Gaafar, M. A. Dual-input configuration of three-phase split-source inverter for photovoltaic systems with independent maximum power point tracking. *Electr. Power Syst. Res.* **232**, 110375 (2024).

[20] Abdelaleem, A., Ismeil, M. A., Nasrallah, M., Mohamed, E. E. & Ali, A. I. M. Advanced control of split source inverter through finite control-set model predictive control for improved system performance. *PloS one* **19**(7), e0305138 (2024). 38985804 10.1371/journal.pone.0305138 PMC11236209

[21] Abdelaleem, A., Ismeil, M. A., Ali, A. I. M., Nasrallah, M., Ali, A., Shabaan, M. F., & Mohamed, E. E. Advancements in topology and modulation techniques for split source inverters: A comprehensive overview. in *2024 6th International Youth Conference on Radio Electronics, Electrical and Power Engineering (REEPE)* 1–8. (IEEE, 2024).

[22] Elthokaby, Y., Abdelsalam, I., Abdel-Rahim, N. & Mohamed, I. Simplified three-phase split-source inverter for PV system application controlled via model-predictive control. *Int. J. Circuit Theory Appl.* **52**(5), 2266–2289 (2024).

[23] Barik, P. K., Samal, S., Gupta, D. K., Appasani, B., Jha, A. V., Islam, M. M., & Ustun, T. S. Split‐source inverter with adaptive control scheme‐based shunt active power filter for power quality improvement. *IET Power Electronics*. (2024).

[24] lotfy, M. W., Dabour, S. M., Mostafa, R. M., Almakhles, D. J., & Elmorshedy, M. F. Modeling and control of a voltage-lift cell split-source inverter with MPPT for photovoltaic systems. *IEEE Access*, **11**, 54699–54712. 10.1109/ACCESS.2023.3280602 (2023).

[25] Abu-Zaher, M., Zhuo, F., Gaafar, M. A., Orabi, M., Hassan, A., & Aly, M. Dual-input single-phase split source inverter for optimized power extraction of grid-connected PV systems under varied atmospheric conditions. in *2024 IEEE 10th International Power Electronics and Motion Control Conference (IPEMC2024-ECCE Asia)* 2929–2934. (IEEE, 2024).

[26] MKP, M. R., & Ahmad, M. W. A robust open circuit fault detection and localization scheme for HERIC PV inverter. in *IEEE Trans. Ind. Electron.*10.1109/TIE.2025.3528481.

[27] El-Hendawy, N., Dabour, S. M. & Rashad, E. M. Common-mode voltage analysis of three-phase quasi-Z source inverters for transformerless photovoltaic systems. *IEEE Conf. Power Electron. Renew. Energy (CPERE)* **2019**, 355–360. 10.1109/CPERE45374.2019.8980256 (2019).

[28] Anand, V., Singh, V. & Sathik, M. J. A. Reduced component high voltage boost single-source switched capacitor inverter. *Sādhanā* **47**, 59. 10.1007/s12046-022-01838-x (2022).

[29] Anand, V., Sathik, J., Garcia, C., Blaabjerg, F. & Rodríguez, J. ANPC switched-capacitor 19L inverter using SHE PWM for 1-ϕ HFAC PDS applications. *IEEE J. Emerg. Sel. Top. Power Electron.* **12**(5), 4494–4505. 10.1109/JESTPE.2024.3432133 (2024).

[30] Abdelhakim, A., Davari, P., Blaabjerg, F. & Mattavelli, P. Switching loss reduction in the three-phase quasi-z-source inverters utilizing modified space vector modulation strategies. *IEEE Trans. Power Electron.* **33**(99), 1–1 (2017).

[31] Anand, V. & Singh, V. A 13-level switched-capacitor multilevel inverter with single DC source. *IEEE J. Emerg. Sel. Top. Power Electron.* **10**(2), 1575–1586. 10.1109/JESTPE.2021.3077604 (2022).

[32] Muhammad, R. H. (ed.). Power electronics handbook. (Butterworth-Heinemann, 2017).

[33] Wageh, M., Dabour, S. M., & Mostafa, R. M. A new four-switch split-source boosting inverter: Analysis and Modulation. in. *23rd International Middle East Power Systems Conference (MEPCON)*. 1–7, 10.1109/MEPCON55441.2022.10021772 (Cairo, Egypt 2022).

[34] Wageh, M., Dabour, S. M., & Mostafa, R. M. A high gain split-source inverter with reduced input current ripple. in *2021 22nd International Middle East Power Systems Conference (MEPCON)*, 383–388, 10.1109/MEPCON50283.2021.9686237. (2021).

[35] Salehizadeh, M., Montazeri, S. H., Milimonfared, J. & Rastegar, H. Modified space vector pulse width modulation for three-phase high voltage gain switched-inductor split-source inverter. *AUT J. Electr. Eng.* **56**(1), 141–150 (2024).

[36] Farhangi, M., Barzegarkhoo, R., Lee, S. S., Aguilera, R. P., Lu, D., & Siwakoti, Y. P. An interleaved single-stage switched-boost common-ground multilevel inverter: design, control, and experimental validation. *IEEE Trans. Ind. Appl* (2024).

[37] Barzegarkhoo, R., Farhangi, M., Lee, S. S., Siwakoti, Y. P., & Blaabjerg, F. Switched-boost-based multilevel inverters. in *Control of Power Electronic Converters and Systems: Volume 4* 127–154. (Academic Press, 2024).

[38] Boscaino, V. et al. Grid-connected photovoltaic inverters: Grid codes, topologies and control techniques. *Renew. Sustain. Energy Rev.* **189**, 113903 (2024).

[39] Lee, S., Kim, Y. J. & Yoo, H. Split-gate: Harnessing gate modulation power in thin-film electronics. *Micromachines* **15**(1), 164 (2024). 38276863 10.3390/mi15010164 PMC10820144

[40] Baier, C. R. et al. Developing and evaluating the operating region of a grid-connected current source inverter from its mathematical model. *Mathematics* **12**(12), 1775 (2024).

[41] Nozadian, M. H. B., Hassanpour, N., & Khan, S. Enhanced switched impedance inverter with tapped inductor. in *2024 IEEE 18th International Conference on Compatibility, Power Electronics and Power Engineering (CPE-POWERENG)* 1–6. (IEEE, 2024).

[42] PEModule Technologies. Available online: www.pemodule.com (Accessed 01 November 2022).

[43] Faraji, F., Hajirayat, A., Birjandi, A. A. M. & Al-Haddad, K. Single-stage single-phase three-level neutral-point-clamped transformerless grid-connected photovoltaic inverters: Topology review. *Renew. Sustain. Energy Rev.* **80**, 197–214 (2017).

[44] Shayestegan, M. et al. An overview on prospects of new generation single-phase transformerless inverters for grid-connected photovoltaic (PV) systems. *Renew. Sustain. Energy Rev.* **82**, 515–530 (2018).

[45] Kihal, A., Talbi, B., Krama, A., Laib, A., & Sahli, A. A multi-functional grid-tied PV system using a split source inverter with energy management and power quality improvement features. *IEEE Access*10.1109/ACCESS.2025.3538069.

[46] Zeb, K. et al. A comprehensive review on inverter topologies and control strategies for grid connected photovoltaic system. *Renew. Sustain. Energy Rev.* **94**, 1120–1141 (2018).

[47] Mahela, O. P. & Shaik, A. G. Comprehensive overview of grid interfaced solar photovoltaic systems. *Renew. Sustain. Energy Rev.* **68**, 316–332 (2017).

[48] Latran, M. B. & Teke, A. Investigation of multilevel multifunctional grid connected inverter topologies and control strategies used in photovoltaic systems. *Renew. Sustain. Energy Rev.* **42**, 361–376 (2015).

[49] Çelik, Ö., Teke, A. & Tan, A. Overview of micro-inverters as a challenging technology in photovoltaic applications. *Renew. Sustain. Energy Rev.* **82**, 3191–3206 (2018).

[50] Gopi, R. R. & Sreejith, S. Converter topologies in photovoltaic applications–A review. *Renew. Sustain. Energy Rev.* **94**, 1–14 (2018).

[51] Lotfy, M. W., Ramadan, H. S. & Dabour. S. M. Smart EV charging via advanced ongrid MPPT-PV systems with quadratic-boost split-source inverters. *Sci. Rep.* **15**(1), 7841. 10.1038/s41598-025-90775-w (2025). 10.1038/s41598-025-90775-w PMC11885632 40050319

[52] Baksi, S. K., Behera, R. K., & Muduli, U. R. A comprehensive analysis of enhanced DC-bus utilization and reduced component count five-level inverter for PV-grid integration. in *IEEE Trans. Ind. Appl.*10.1109/TIA.2025.3532231.

[53] Anand, V., Singh, V., Sathik, J. & Almakhles, D. Single-stage five-level common ground transformerless inverter with extendable structure for centralized photovoltaics. *CSEE J. Power Energy Syst.* **9**(1), 37–49. 10.17775/CSEEJPES.2022.02700 (2023).

[54] Anand, V. et al. Seventeen level switched capacitor inverters with the capability of high voltage gain and low inrush current. *IEEE J. Emerg. Sel. Top. Ind. Electron.* **4**(4), 1138–1150. 10.1109/JESTIE.2023.3291996 (2023).

[55] Bašić, M., Vukadinović, D., & Grgić, I. Mitigating dead-time impact in a three-phase split-source inverter: A simple compensation method. in *2024 23rd International Symposium INFOTEH-JAHORINA (INFOTEH)* 1–6. (IEEE, 2024).

